# Cognitive and perceptual load have opposing effects on brain network efficiency and behavioral variability in ADHD

**DOI:** 10.1162/netn_a_00336

**Published:** 2023-12-22

**Authors:** Jacob T. Fisher, Frederic R. Hopp, René Weber

**Affiliations:** Department of Communication, Michigan State University, East Lansing, MI, USA; Amsterdam School of Communication Research, University of Amsterdam, Amsterdam, Netherlands; Department of Communication, Media Neuroscience Lab, University of California Santa Barbara, Santa Barbara, CA, USA; Department of Psychological and Brain Sciences, University of California Santa Barbara, Santa Barbara, CA, USA; School of Communication and Media, Ewha Womans University, Seoul, South Korea

**Keywords:** ADHD, fMRI, Cognitive load, Perceptual load, Brain networks

## Abstract

Attention-deficit/hyperactivity disorder (ADHD) is a highly prevalent neurodevelopmental disorder associated with suboptimal outcomes throughout the life-span. Extant work suggests that ADHD-related deficits in task performance may be magnified under high cognitive load and minimized under high perceptual load, but these effects have yet to be systematically examined, and the neural mechanisms that undergird these effects are as yet unknown. Herein, we report results from three experiments investigating how performance in ADHD is modulated by cognitive load and perceptual load during a naturalistic task. Results indicate that cognitive load and perceptual load influence task performance, reaction time variability (RTV), and brain network topology in an ADHD-specific fashion. Increasing cognitive load resulted in reduced performance, greater RTV, and reduced brain network efficiency in individuals with ADHD relative to those without. In contrast, increased perceptual load led to relatively greater performance, reduced RTV, and greater brain network efficiency in ADHD. These results provide converging evidence that brain network efficiency and intraindividual variability in ADHD are modulated by both cognitive and perceptual load during naturalistic task performance.

## Author Summary

Attention-deficit/hyperactivity disorder (ADHD) affects more than 500 million adults worldwide (Song et al., 2021), and confers suboptimal outcomes in many areas of life. Emerging evidence suggests that ADHD-related cognitive deficits may be at least somewhat task-dependent, with some work showing that increased cognitive load results in reduced performance in ADHD relative to non-ADHD individuals, and other work showing that increased perceptual load may narrow, or even eliminate these deficits. However, these effects have yet to be examined together, and their neural underpinnings are unknown. In three experiments (one in the lab, one online, and one fMRI), we show that cognitive load worsens cognitive performance, and reduces brain network efficiency in ADHD relative to non-ADHD individuals, while perceptual load has largely the opposite effect.

## INTRODUCTION

Attention-deficit/hyperactivity disorder (ADHD) is a highly prevalent cognitive processing disorder that confers suboptimal outcomes in many areas of life, including reduced educational and vocational achievement (Biederman et al., 2004), higher rates of drug and alcohol abuse (Biederman et al., 1998), lower self-esteem (Harpin et al., 2016), and diminished overall well-being (Danckaerts et al., 2010). One of the most stable and universal features of the ADHD phenotype is high intraindividual variability in behavior (Kofler et al., 2013; Tamm et al., 2012). Individuals with ADHD frequently exhibit large fluctuations in reaction time and accuracy during task performance (Kofler et al., 2013; Tamm et al., 2012). These fluctuations are theorized to result from reduced information processing efficiency in the ADHD brain (Russell et al., 2006). Supporting this hypothesis, recent evidence shows that elevated reaction time variability (RTV) is associated with less efficient connectivity patterns in the brain (Machida et al., 2019), and that those with ADHD exhibit reduced brain network efficiency both at rest and during cognitive tasks (Konrad & Eickhoff, 2010).

There is mounting evidence that clinically relevant individual differences can be modulated by carefully chosen task parameters, enabling researchers to design tasks that maximize (or minimize) these differences to highlight specific cognitive and neural processes implicated in mental disorders (Finn et al., 2017, 2020). Comparatively naturalistic (e.g., smoothly evolving in time and perceptually engaging) tasks such as movies and video games have proven especially useful in this endeavor, serving to constrain undesirable confounds (like head motion and mind wandering) while generating rich, time-locked neural activity patterns not observable in trial-based neuroimaging paradigms (Vanderwal et al., 2017). These efforts have increased understanding of a number of mental disorders, including autism (Bolton et al., 2018), paranoia (Finn et al., 2018), and depression (Guo et al., 2015).

In the present study, we use a custom-developed and validated video game task^1^ (Huskey et al., 2018) to investigate cognitive control performance and brain network efficiency in ADHD across three experiments (one in a computer lab, one online, and one fMRI). The task’s primary goal consisted of collecting objects (crystals) while dodging asteroids. Participants completed three variants of the video game task, one with increased cognitive load, one with increased perceptual load, and one containing neither the cognitive nor the perceptual load manipulations. Extant work suggests that cognitive load and perceptual load may differentially impact task performance in ADHD. Cognitive load has been shown to magnify ADHD-related performance deficits, leading to comparatively worse performance in those with ADHD (Roberts et al., 2012). In contrast, perceptual load has been shown to minimize, or even eliminate, performance gaps between those with and without ADHD (Forster et al., 2014; Forster & Lavie, 2007). As such, we expected that increasing cognitive load would have a disproportionately negative influence on task performance and brain network efficiency in ADHD, and that increasing perceptual load would have the opposite effect, reducing the gap between ADHD and non-ADHD groups in both performance and brain network efficiency.

## MATERIALS AND METHODS

### General Overview

**Figure 1.**
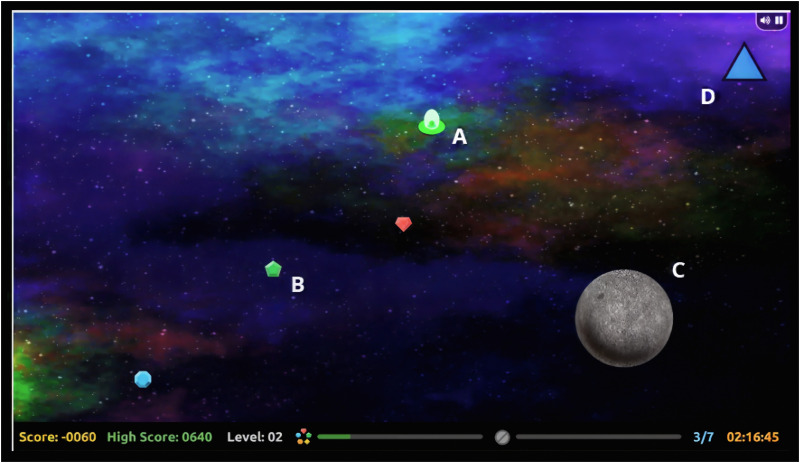
Schematic of Asteroid Impact gameplay. The primary goal of the game is to navigate a “spaceship” (A) around the screen to collect as many crystals (B) as possible before time runs out, while concurrently avoiding asteroids (C) and responding to intermittent speeded reaction time probes (D). Asteroid Impact adapts to the skill level of the player by increasing the quantity, size, and speed of asteroids, ensuring that all participants play at a difficulty level that is approximately equal to their skill. This serves to minimize differences in performance between players as a result of skill.

All research was conducted in accordance with the Human Subjects Committee of the university (IRB Protocol removed for anonymous review). In each of the three presented experiments, we used *Asteroid Impact*, an open-source video game task.^2^ In the first two experiments, participants played *Asteroid Impact* in a lab environment, and in the third, participants played a browser-based version on their personal computer at home. The primary goal in *Asteroid Impact* is to navigate a spaceship around the screen with the mouse, collecting valuable crystals while avoiding asteroids (see Figure 1). Previous work has shown that cognitive resource availability tends to be high during boredom (ability >> difficulty) and frustration (ability << difficulty), and low whenever difficulty and ability are approximately matched (Harris et al., 2017; Huskey et al., 2018). As such, we designed the game to adapt its difficulty level (the speed, quantity, and size of asteroids) to player performance over time, ensuring that all participants are challenged at a level that meets (but does not exceed) their abilities. *Asteroid Impact* has been previously shown to be motivating and engaging, and to be usable within both a behavioral and fMRI setting (Huskey et al., 2018).

In each of the three experiments, participants played three different variations of the game: a baseline condition, a cognitive load condition, and a perceptual load condition. These conditions were identical across the three experiments. Participants also completed a speeded reaction time task during gameplay, in which they were asked to press the “X” key on a keyboard when they saw a blue square appear on screen, and to press “Y” when they saw a purple triangle. This task was designed to index the speed and variability in participants’ response times during each condition. In each experiment, the speeded reaction time task was rewarded in-game points. The triangle was worth 10 points, and the square was worth 1,000 points. In all experiments reported herein, we only consider reaction time data from the low-reward probes, as reward is known to modulate both the speed and variability of response times in ADHD (Tamm et al., 2012).

#### Manipulating cognitive load.

Cognitive load was introduced into the game using a variant of an *n*-back rule maintenance task. The *n*-back is a widely used experimental paradigm in the fields of neuroscience and cognitive psychology in which individuals must perform a particular action—typically pressing a button—whenever a given stimulus matches a stimulus that occurred a certain number of trials in the past (Owen et al., 2005). This manipulation has been shown to elicit activation in working memory related brain regions and to be perceived as cognitively difficult (Eriksson et al., 2015). In the version of the *n*-back tasks employed in this study, an instruction screen informed participants that “some of the crystals are sabotaged” and that collecting two crystals of the same color in a row would result in a loss of 1,000 in-game points. This required participants to maintain the identity of the most recently collected crystal in working memory while collecting the next crystal.

#### Manipulating perceptual load.

The study of perceptual load has a rather long and contentious history within visual perception and cognitive neuroscience research (Fitousi & Wenger, 2011), but a recurring finding is that increased perceptual load can improve performance in visual perception and cognitive control tasks in people with ADHD (see, e.g., Forster et al., 2014). Perceptual load has been manipulated in several ways, including increasing the number of items that need to be identified at any given time, increasing the difficulty of item identification (through blurring, distortion, rotation, etc.), increasing auditory or visual background noise, and reducing contrast between the foreground and the background (Elliott & Giesbrecht, 2010). In this study, we manipulated perceptual load by adding an overlay to the gameplay environment that reduced the opacity of foreground elements (asteroids and crystals) by 75% relative to the background.

#### Measuring performance.

As the primary goal in *Asteroid Impact* is to collect crystals while avoiding asteroids, we measured in-game performance as the number of crystals collected within a 30-second window divided by one plus the number of asteroid collisions within the same window.^3^ Mean performance did not significantly vary between Experiment 1 and Experiment 3 (*M*_*S1*_ = 30.4, *M*_*S3*_ = 31.2), but was lower in Experiment 2 (*M*_*S2*_ = 18.61), perhaps due to the unfamiliarity of the controls used in the fMRI experiment. RTV was measured by fitting an ex-Gaussian distribution to each participant’s reaction times within each round and taking the standard deviation of reaction times falling within the exponential portion of the distribution. Overall RTV was higher in Experiment 3 (*M* = 1,241.96) than in Experiment 1 (*M* = 962.26) or Experiment 2 (*M* = 987.92), likely due to the online (i.e., less controlled) nature of the experiment environment.

### Experiment 1

#### Participants.

Participants in Experiment 1 were recruited from the participant pool of the Communication Department at a large university in the western United States. All participants provided written informed consent and earned course credit for their participation. In total, 230 participants completed the experiment (159 female, 69 male, 2 chose not to answer, *M*_*age*_ = 19.58). Two participants reported having taken medication for ADHD within the 12-hour period before their session, and as such were excluded from the analyses reported herein.

#### Experimental design.

Participants were invited into a computer lab with ten cubicles, each containing a Dell computer with a 16 × 9 inch monitor. Each participant was given a consent form outlining the purpose of the experiment and describing the *Asteroid Impact* task. After the participants finished reading and signing the consent forms, a researcher read a short prompt reiterating the goal of the game along with its controls. After this, participants put on headphones to minimize distraction, and began playing the game. Participants completed seven rounds of gameplay in total—a practice round and two rounds in each condition. All rounds following the practice round were presented in randomized order. After all levels were completed, participants completed the questionnaire items and were dismissed and thanked for their participation.

ADHD symptom severity was determined using the full version of the Adult ADHD Self-Report Scale (ASRS; Kessler et al., 2005). The ASRS has been shown to have 97.9% total classification accuracy (sensitivity 68.7%) for clinicians’ ADHD diagnosis, a *κ* of .76, and a Chronbach’s ⍺ in between .63 and .72 in the general population of the United States (Kessler et al., 2007). ASRS scores range from 1 to 4. Participants who scored 3.2 or greater (1 standard deviation above the mean) were considered as high symptom severity (*N* = 54), and those who scored 2.0 or lower (1 standard deviation below the mean) were considered as low symptom severity (*N* = 48). In addition to the ASRS, participants completed a series of self-report questionnaires and provided basic demographic data. Data from these additional questionnaires are not reported herein.

#### Analyses.

All analyses in Experiment 1 were conducted in R (R Core Team, 2013). Before analysis, data were minimally preprocessed, removing RTs that were greater than 5 standard deviations away from the mean within subjects and conditions. All main effects were tested using linear mixed-effects models using the *lmer()* function from the *lme4* package in R (Bates et al., 2015). Cognitive load, perceptual load, and ADHD symptom severity were treated as fixed effects, and were coded using effects coding. Random intercepts were included for each participant. All reported betas are standardized. Correction for multiple comparisons was conducted using the Tukey method. As an additional check, we also conducted these analyses in a manner treating ASRS scores as a continuous variable rather than binning participants and only retaining high and low-ADHD groups. These analyses can be found in Supporting Information Figure S2.

#### Results.

**Figure 2.**
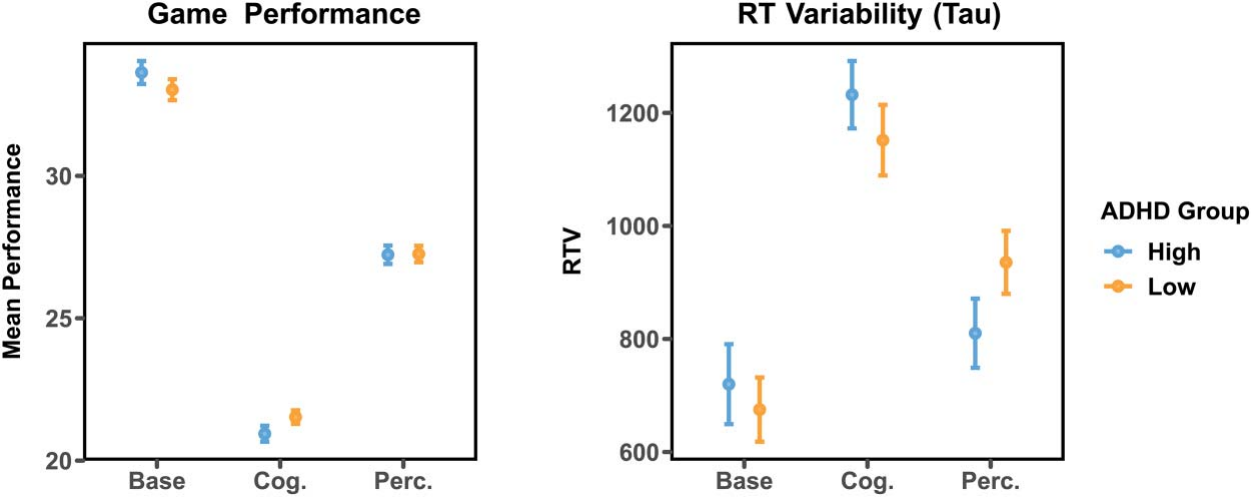
Task performance (crystals collected/asteroid collisions) and reaction time (RT) variability in high-ADHD and low-ADHD groups during the baseline, cognitive load, and perceptual load conditions in Experiment 1 (in-lab). Error bars represent 95% confidence intervals (within subjects).

Results from Experiment 1 indicate that cognitive and perceptual load impact behavioral variability and task performance in a manner contingent on ADHD symptoms (see Figure 2). Under high perceptual load, the group with high levels of ADHD symptoms had lower RTV—an indicator of fewer attentional lapses during game play—than did the low-ADHD group (*M*_*hi*_ = 709.99, *SD* = 432.03 *M*_*lo*_ = 735.64, *SD* = 518.99), and the interaction between ADHD status and load condition on RTV was statistically significant (*F*(1, 226) = 6.97, *p* = .009). Those with high-ADHD symptom severity also underperformed in the crystal collection task during the cognitive load condition compared to those with mild or nonexistent ADHD symptoms (*M*_*hi*_ = 20.78, *SD* = 5.33; *M*_*lo*_ = 21.67, *SD* = 4.52), again with a significant interaction between ADHD status and load condition (*F*(1, 226) = 5.54, *p* = .018). This pattern was not observed in the baseline or perceptual load conditions.

### Experiment 2

#### Participants.

Participants in this experiment were recruited from the participant pool of the Communication Department at a large university in the western United States and from the general community surrounding the university. A total of 36 participants (23 female, 13 male, *M*_*age*_ = 19.76) were recruited—18 participants with high-ADHD symptom severity and 18 participants with low-ADHD symptom severity. Participants were recruited using a survey prescreener with the same cutoff points as were used in Experiment 1—ASRS ≥ 3.2 for the high-ADHD group and ASRS ≤ 2.0 for the low-ADHD group. Those whose ASRS scores were above the high-ADHD cutoff or below the low-ADHD cutoff were contacted by a researcher and invited to schedule a brain imaging appointment. Participants were asked to refrain from taking any ADHD medication for at least 12 hours before their appointment. Data from two participants were excluded due to equipment malfunction.

#### Scanning parameters and preprocessing.

All brain imaging data were collected on a 3T Siemens Magnetom Prisma (TR = 400 ms, TE = 35 ms, flip angle = 52°, acquisition matrix = 64 × 64, in-plane resolution = 3 mm^3^). Upon arriving in the Brain Imaging Center, participants provided informed consent, and filled out a metal-screening form. After this, participants spent approximately 10 minutes seated at a laptop practicing the video game task that they would be performing in the scanner. Upon completing the practice task, participants changed into scrubs and a researcher positioned them in the scanner, where they underwent a T1 structural scan followed by the video game task, a T2 structural scan, and a short gambling task which is not reported here. Functional runs included three 600-volume repetitions within each condition (baseline, cognitive load, perceptual load).

All brain imaging data were preprocessed with *fMRIprep*, a Nipype based tool (Esteban et al., 2019; Gorgolewski et al., 2011), and with *xcpEngine*, a supplemental pipeline for denoising data used in functional connectivity analyses (Ciric et al., 2017; Lydon-Staley et al., 2019). Each T1w volume was corrected for intensity nonuniformity using N4 bias field correction from the ANTs registration suite (Avants et al., 2011), and then skull-stripped using the OASIS template provided by ANTs. Brain surfaces were reconstructed using FreeSurfer (Dale et al., 1999), and spatially normalized to the ICBM 152 nonlinear asymmetrical template (version 2009c) using ANTs. Brain tissue segmentation was performed using FSL FAST (Jenkinson et al., 2012). Functional data were slice time corrected using the 3dTShift function from the AFNI software package (Cox, 1996) and motion corrected using FSL MCFLIRT. Following this, functional data were coregistered to the T1w anatomical image using boundary-based registration with 6 degrees of freedom (Greve & Fischl, 2009). Motion correcting transformations, BOLD to T1w transformation, and registration of the T1w to the MNI template were conducted using ANTs. ICA-based AROMA was used to generate aggressive noise regressors and to create output files that are nonaggressively denoised. Further preprocessing conducted in xcpEngine included denoising time series data based on the ICA-AROMA and global signal, and white matter confounds generated by fMRIPrep, and conducting temporal band-pass filtering (0.01 < f < 0.15 Hz) to reduce the influence of low-frequency drift and high-frequency physiological noise.

#### Network creation and analysis.

**Figure 3.**
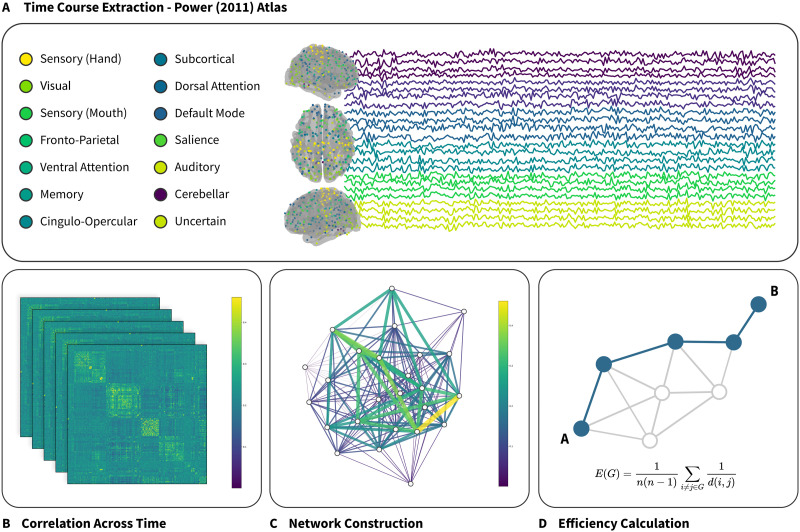
Conceptual depiction of brain network extraction and analysis. (A) Time series data is averaged within a 5-mm sphere surrounding each of 264 regions of interest (ROIs) as defined in the Power et al. (2011) atlas. This atlas is divided into 14 subnetworks as defined by task-based functional connectivity. (B) Pairwise correlation is calculated between each of the 264 ROIs, resulting in a 264 × 264 correlation matrix. (C) Edge weights in the network are assigned as the Pearson’s *R* value between each node. (D) Network efficiency is calculated as the inverse of the mean of all pairwise shortest paths.

After preprocessing, each run was divided into eight nonoverlapping segments of 75 TRs, and the mean signal was extracted from the prewhitened time series in each of 264 regions of interest (ROIs) as defined in Power et al. (2011). Edges were constructed pairwise between each node with edge weights assigned as the correlation between the time series extracted from each pair of regions, resulting in a matrix of Pearson’s R values (see Figure 3). Network efficiency was calculated within each chunk as the inverse of the average shortest path distance between each pair of nodes in the whole-brain network (*M* = .19, *SD* = .012). Following previous studies (see, e.g., Lydon-Staley et al., 2019), we only considered positively weighted edges, setting all negative edges to zero. Efficiency scores between high-ADHD and low-ADHD groups in each condition were compared using a linear mixed-effects model fit using the lmer() function from the *lme4* package in R. ADHD status and experimental condition were treated as fixed effects. Random intercepts and slopes were included for each participant and for each condition, run, and segment nested within each participant.

#### Results.

**Figure 4.**
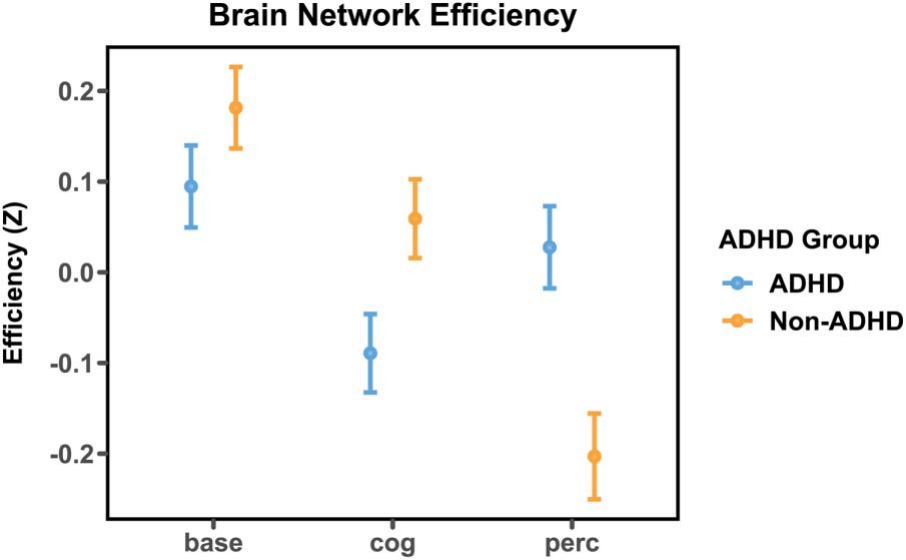
Global efficiency (normalized) under baseline, cognitive load, and perceptual load conditions. During cognitive load, those with ADHD exhibited lower efficiency values than those without ADHD, whereas under perceptual load the opposite pattern was observed.

In the second experiment, we sought to elucidate a candidate neural mechanism for the ADHD-specific influence of cognitive and perceptual load we observed in Experiment 1. Of primary interest was *global efficiency*—the inverse of the average shortest path length between nodes in the whole-brain functional connectivity network. Results show that under cognitive load, those in the high-ADHD group had lower global efficiency than the low-ADHD group, but that this pattern was reversed in the perceptual load condition (see Figure 4). These efficiency differences that we observed between high-ADHD and low-ADHD groups seem to be driven by varying patterns of connectivity in attention-related brain networks, including the fronto-parietal control network, the salience network, and the ventral attention network (see Supporting Information Figure S4; https://osf.io/9byvq/?view_only=49b1b33ec5c14766b534f57d3d8606f2). Furthermore, we observed that the relationship between brain network efficiency and RTV was contingent upon task condition. Increased global efficiency was associated with higher RTV in the baseline and perceptual load conditions, (*β* = .025, *p* < .001), but was associated with lower RTV in the cognitive load condition (*β* = −.17, *p* < .001), suggesting that increased RTV during cognitive load in those with high-ADHD symptoms may be at least partially attributable to a decrease in network efficiency in the brain.

### Experiment 3

#### Participants.

Participants in Experiment 3 were recruited using an online sample from the online service Prolific Academic. In total, 825 participants completed the prescreener for the experiment, for which they earned $5. Participants who self-reported an ADHD diagnosis, and who also met the minimum symptom severity thresholds established in Experiment 1 were invited to complete the second half of the experiment, along with a matched group of those who reported having never been diagnosed with ADHD, and whose reported ADHD symptom severity was below the cutoff outlined above. As in Experiment 2, participants were asked to refrain from taking their ADHD medication for at least 12 hours before participating. As an additional check, participants were also asked to report if they had taken their medication within the last 12 hours, and none reported having done so. One hundred participants completed the second half of the experiment (50 ADHD, 50 non-ADHD, 36 female, 64 male, *M*_*age*_ = 30.22). All participants provided informed consent and earned an additional $5 for their participation in the second half of the experiment.

#### Experimental design.

Participants played a browser-based (WebGL) version of *Asteroid Impact* created using the Unity game design software. In order to ensure minimal lag in reporting reaction times and other in-game events due to internet speeds and ping times, all game logging was performed on participants’ local machines, and then uploaded at the end of each round to a server. Participants were required to complete the game on a personal computer with a screen at least 11” in size, and that has a mouse pointer rather than a touchscreen interface. Participants completed seven rounds of gameplay in total—a 1-minute practice round and two 3-minute rounds in each condition. All rounds following the practice round were presented in randomized order. After all levels were completed, participants were thanked for their submission and dismissed.

#### Analysis.

As in Experiment 1, all analyses were conducted in R and all data were subjected to minimal preprocessing. All main effects were tested using linear mixed-effects models. Cognitive load, perceptual load, and ADHD diagnosis were treated as fixed effects, and were coded using effects coding. Random intercepts were included for each participant. All reported betas are standardized. As in Experiment 1, correction for multiple comparisons was conducted using the Tukey method.

#### Results.

**Figure 5.**
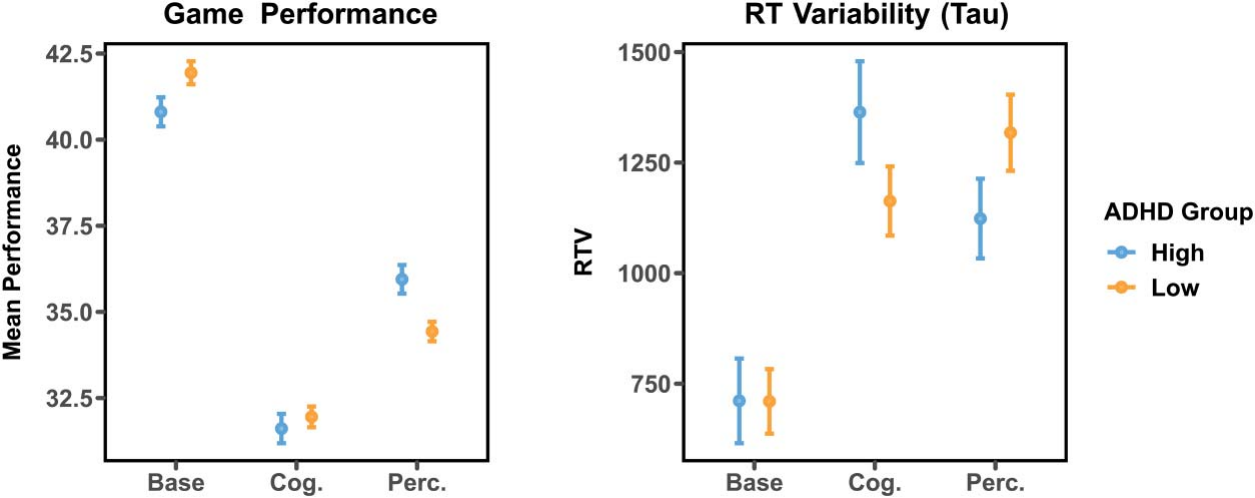
Task performance (crystals collected/asteroid collisions) and reaction time (RT) variability in high-ADHD and low-ADHD groups during the baseline, cognitive load, and perceptual load conditions in Experiment 3 (online). Error bars represent 95% confidence intervals (within subjects).

In the third experiment, (*N* = 100) we extended the findings of Experiments 1 and 2, recruiting a nonstudent sample and conducting the experiment in a more ecologically valid setting—online, on participants’ personal computers in their own homes. To do so, we developed an online version of the video game task used in experiment one and two. We again observed an ADHD-specific influence of both cognitive and perceptual load on both RTV (*F*(1, 98) = 3.67, *p* = .03) and task performance (*F*(1, 98) = 39.88, *p* < .001, see Figure 5). Replicating Experiment 1, those with ADHD exhibited lower RTV than those without ADHD during perceptual load (*M*_*ADHD*_ = 795.07, *M*_*N-ADHD*_ = 937.91). The opposite pattern was observed during cognitive load, in which those with ADHD exhibited higher RTV than those without ADHD (*M*_*ADHD*_ = 1,114.51, *M*_*N-ADHD*_ = 928.26). As in experiment one, those with ADHD underperformed compared to those without ADHD during the baseline condition (*M*_*ADHD*_ = 40.97, *M*_*N-ADHD*_ = 41.92), and outperformed those without ADHD during perceptual load (*M*_*ADHD*_ = 35.71, *M*_*N-ADHD*_ = 34.41).

## DISCUSSION

In the present study, we provide evidence that cognitive control performance in ADHD during a naturalistic task is contingent on both cognitive load and perceptual load. Cognitive load had a detrimental impact on performance, RTV, and brain network efficiency across both ADHD and non-ADHD groups, but it had a disproportionately negative influence on those with ADHD, widening the gap in performance between non-ADHD and ADHD groups. In contrast, perceptual load eliminated differences in brain network efficiency between ADHD and non-ADHD groups, and in some cases driving the ADHD group to perform better and respond less variably to reaction time probes than the non-ADHD group. Our results provide evidence for the task-dependent nature of ADHD-related neural and behavioral differences and highlight the usefulness of a “modding” approach to incorporate controlled manipulations into a naturalistic task. These results reveal rich information regarding when and why ADHD individuals may perform less optimally than those without ADHD, and how performance in ADHD may be improved by modifying the parameters of a task.

### Cognitive Load Magnifies Behavioral and Neural Indicators of ADHD Symptoms

Previous studies have focused on the distraction-magnifying influence of cognitive load in attention-demanding tasks (Kelley & Lavie, 2011), and have suggested that differential susceptibility to cognitive load (e.g., via reduced working memory capacity) may be linked to suboptimal cognitive performance in those with ADHD (Roberts et al., 2012). In the present study, we demonstrate that increased cognitive load has a detrimental influence on task performance and RTV in both ADHD and non-ADHD individuals, but that those with ADHD are comparatively more affected. We also show that this relationship is observable under both categorical and continuous characterizations of ADHD symptoms. During the cognitive load condition, those with ADHD performed worse in the video game task, had higher RTV, and had lower brain network efficiency than those without ADHD.

Our results regarding brain network efficiency can also be interpreted in light of recurrent observations that those with ADHD exhibit decreased efficiency in resting state and task-evoked brain networks (for a review, see Konrad & Eickhoff, 2010), as well as aberrant modulation of default mode and fronto-parietal attention networks (Castellanos & Proal, 2012). These modulations have also been linked to trial-to-trial variation in attentional lapses (Prado et al., 2011). Increased global efficiency in functional brain networks has been linked with greater performance in cognitive tasks (Hearne et al., 2017). As such, reconfiguration of global brain networks in those with ADHD in response to increasing cognitive load could be expected to result in less efficient network topologies—and to result in decreased performance in the task.

Although lower performance in ADHD has previously been observed in highly controlled cognitive tasks (such as dual choice or flanker tasks), this study is the first to show an ADHD-specific influence of cognitive load in comparatively more “real-world” task performance, and to link differences in performance under cognitive load to variations in brain network efficiency, a putative mechanism in multiple models of ADHD (e.g., Castellanos & Aoki, 2016). Connecting aberrant patterns of functional connectivity with symptom domains is a fundamental question within ADHD research (Castellanos & Proal, 2012). Our observation that those with ADHD perform worse and have lower brain network efficiency under cognitive load suggests that linking naturalistic task performance with indices of brain network topology could provide informative neurobiological signatures distinguishing clinically relevant features of ADHD and predicting both symptoms and responsivity to treatment in these individuals.

### Perceptual Load Minimizes ADHD-Related Performance Deficits and Behavioral Variability

A growing body of research demonstrates that perceptual load may be beneficial to cognitive performance in ADHD when compared to non-ADHD groups (Forster et al., 2014). It has remained unclear, though, whether the benefits of increased perceptual load for those with ADHD are limited to basic, highly controlled cognitive tasks, or whether they are observable in more naturalistic task performance. Here, we demonstrate that increased perceptual load during video game play has a positive influence on both performance and RTV in those with ADHD. Whereas those with ADHD underperformed those without ADHD under cognitive load, they usually outperformed those without ADHD under perceptual load. Our results also provide evidence that intraindividual RTV—a consistently observed ADHD phenotype (Kofler et al., 2013; Tamm et al., 2012)—is modulated by perceptual load. Individuals with ADHD were found to be more variable than those without ADHD during cognitive load but were less variable under perceptual load.

These findings augment recent work suggesting that perceptual load may act to eliminate variability in ADHD attributable to cognitive control deficits (Forster et al., 2014). The Load Theory of Selective Attention and Cognitive Control (Lavie et al., 2004) suggests that perceptual load shifts the balance of attentional selection mechanisms away from executive control and toward more bottom-up filtering and selection. It follows, then, that the addition of perceptual load may enable those with ADHD to leverage relative strengths in sensory integration and perception abilities while minimizing their reliance on cognitive control mechanisms. Indeed, ADHD is associated with increased local efficiency in brain networks (Lin et al., 2014), a topological signature associated with increased performance in perceptual discrimination tasks (Weiss et al., 2011). Further supporting this conclusion is recent work showing that the addition of conflicting dialog (increasing cognitive load) within a naturalistic, multitalker conversation desynchronizes brain activity between individuals with ADHD, but that introducing white noise (perceptual load) does not (Salmi et al., 2020).

Neurobiologically informed video games have recently been introduced as potential interventions for improving ADHD symptoms (Mishra et al., 2016), highlighting neural signatures of distractor suppression and interference resolution as useful targets for intervention. Related work shows that those who habitually play action video games seem to exhibit increased attention, cognitive control, and working memory abilities compared to their peers (see, e.g., Cardoso-Leite et al., 2016; Green & Bavelier, 2003). In contrast, sustained engagement in other media is associated with decreased performance in these same domains. Those who frequently engage in media multitasking (concurrently doing two or more media tasks) exhibit reduced cognitive control and have higher self-reported symptoms of ADHD (Uncapher et al., 2016). These findings highlight the necessity of developing better understanding regarding how individual differences in media habits influence (and are influenced by) cognitive individual differences.

### Conclusions

In three experiments, we showed that ADHD-specific variation in task performance and functional connectivity is modulated by both cognitive load and perceptual load. Cognitive load disproportionately degrades performance, RTV, and brain network efficiency in ADHD, whereas perceptual load has largely opposite effects—minimizing observable differences between ADHD and non-ADHD. These results demonstrate that performance and neural efficiency gaps between ADHD and non-ADHD groups can be minimized or even eliminated when task parameters are changed. This work aligns with a growing collection of evidence showing that changes in cognition and attention during task performance are reflected in changes in brain network topology and dynamics (Rosenberg et al., 2020), and that these patterns of variations can reveal information about underlying clinical conditions that is largely inaccessible using other methods (Finn et al., 2018; Guo et al., 2015; Salmi et al., 2020). Taken together, our findings indicate a number of future research opportunities at the intersection of neuroscience and media design that leverage video game “modding” to generate naturalistic tasks that nonetheless vary along theoretically relevant continua. This work stands to contribute to greater understanding of complex disorders like ADHD and to the development of targeted interventions to improve cognitive performance.

## ACKNOWLEDGMENTS

We thank Jonathan Kim, Rachel Sun, Christian Quebral, and Thea Yodice for their assistance with data collection, Mario Mendoza for assisting with design and implementation of the fMRI protocol, and Scott Grafton for valuable feedback during the preparation of the manuscript.

## SUPPORTING INFORMATION

Supporting information for this article is available at https://doi.org/10.1162/netn_a_00336. The data, code, and materials for all experiments are publicly accessible at https://osf.io/9byvq and at https://github.com/medianeuroscience/asteroid_impact. There is no preregistration for this study.

## AUTHOR CONTRIBUTIONS

Jacob T. Fisher: Conceptualization; Data curation; Formal analysis; Investigation; Methodology; Software; Visualization; Writing – original draft. Frederic R. Hopp: Validation; Writing – review & editing. René Weber: Conceptualization; Formal analysis; Funding acquisition; Methodology; Project administration; Resources; Supervision; Validation; Writing – review & editing.

## FUNDING INFORMATION

R. W. and J. T. F. were supported by the Rutherford Fett Brain Imaging Center Research Fund, the UCSB Faculty Senate Research Grant, and the NSF IGERT Innovation Fund.

## Notes


^1 ^
https://github.com/medianeuroscience/asteroid_impact.
^2 ^
https://github.com/asteroidimpact/asteroid_impact_py3.
^3 ^
One was added to the denominator to avoid dividing by zero.

## TECHNICAL TERMS

ADHD: Attention-deficit/hyperactivity disorder. A highly prevalent neurodevelopmental disorder associated with suboptimal outcomes throughout the life-span. Individuals with ADHD may have trouble concentrating, often act on impulse, and seem restless.
Cognitive load: Cognitive load refers to the amount of information that humans’ cognitive system (especially human’s working memory capacity) can process at any given time.
Perceptual load: Perceptual load refers to the complexity of physical stimuli, especially distractor stimuli. For instance, stimuli with different levels of transparency lead to higher perceptual load in processing than opaque stimuli.
Asteroid Impact: An open-source experimental video game task developed by the Media Neuroscience Lab at the University of California Santa Barbara.
