# Supplementary material for: Cognitive and perceptual load have opposing effects on brain network efficiency and behavioral variability in ADHD
Source: Netw Neurosci. 2023 Dec 22;7(4):1483–96. doi: 10.1162/netn_a_00336 (PMC10727773; doi:10.1162/netn_a_00336)
Supplement: Supplementary file 1 [file netn-7-4-1483-s001.pdf]

Fisher, J.T., Hopp, F.R. & Weber, R. (2023). Cognitive and Perceptual Load Have Opposing Effects on Brain Network Efficiency and Behavioral Variability in ADHD. *Network Neuroscience*, Advance publication. https://doi.org/10.1162/netn_a_00336.

Supplemental Material

Methods

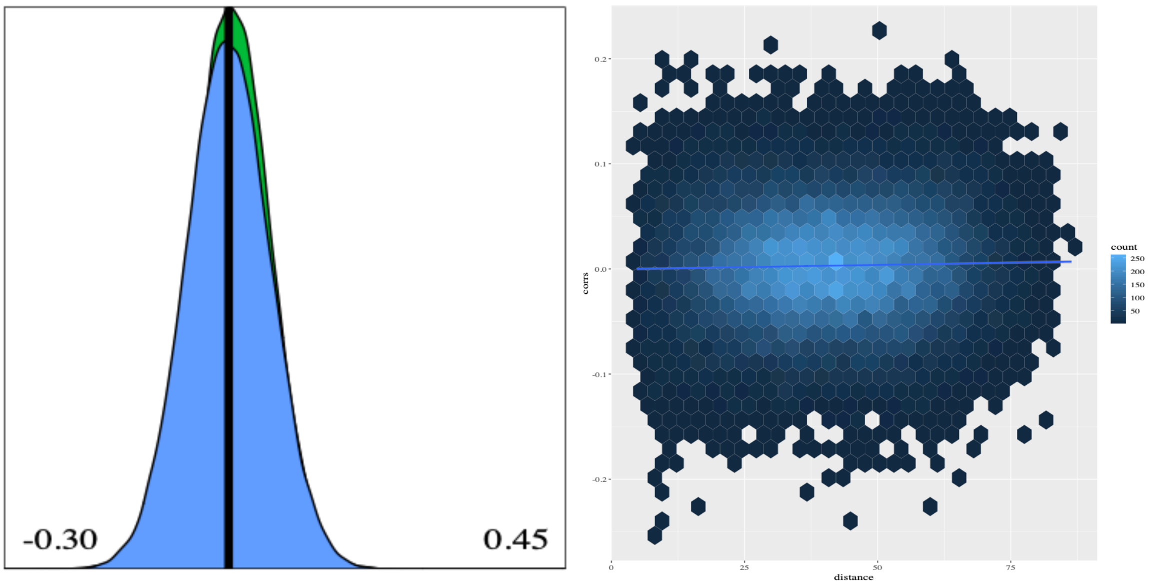

Fig. S1. QC-FC results after denoising with *fMRIPrep* and *xcpEngine.* Absolute median QC-FC correlation = -.03.

Experiment	Group	M_{Age}	% Female	M_{ASRS}
Experiment 1	ADHD	19.75	70.91	3.56
	Non-ADHD	19.39	65.57	2.17
	Overall	19.59	71.54	2.81
Experiment 2	ADHD	19.50	61.11	3.54
	Non-ADHD	20.12	66.67	1.89
	Overall	19.76	63.89	2.87
Experiment 3	ADHD	27.88	40.00	3.83
	Non-ADHD	31.73	33.34	1.61
	Overall	30.22	35.95	2.48

Tab. S1. Participant characteristics in each of the three experiments.

Results

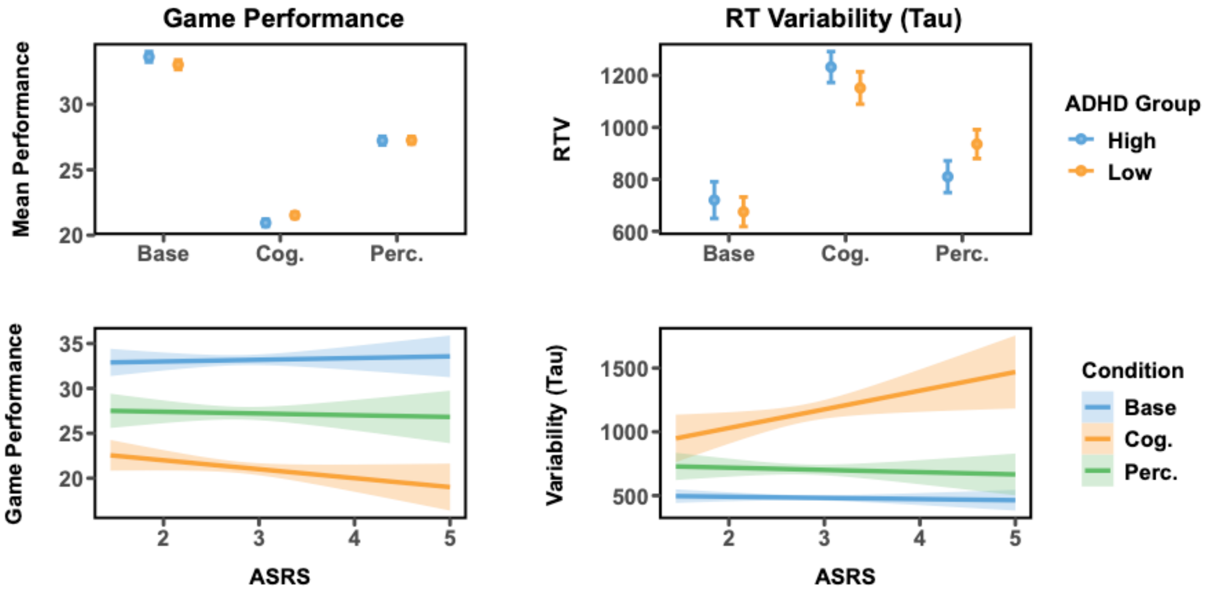

Fig. S2. Analysis of the relationship between ADHD symptom severity (as measured by the ASRS) with game performance (left), and reaction time variability (right). The top two figures show the analysis reported in the main manuscript, in which participants were binned into high and low ADHD groups based on their ASRS scores. The bottom two figures show the same analysis, but with all participants retained and ASRS treated as a continuous variable. An interaction was observed between condition and ADHD symptom severity for performance (F(2, 226) = 4.99, p = .02) such that symptom severity was negatively correlated with performance in the cognitive load condition (β = -.54, p = .009), but not in the perceptual load condition (β = -.10, p = .77) or the baseline condition (β = .10, p = .71). Although the correlation between reaction time variability was positive in the cognitive load condition (β = .36), and negative in the perceptual load condition (β = -.07), the interaction was not statistically significant (p = .17).

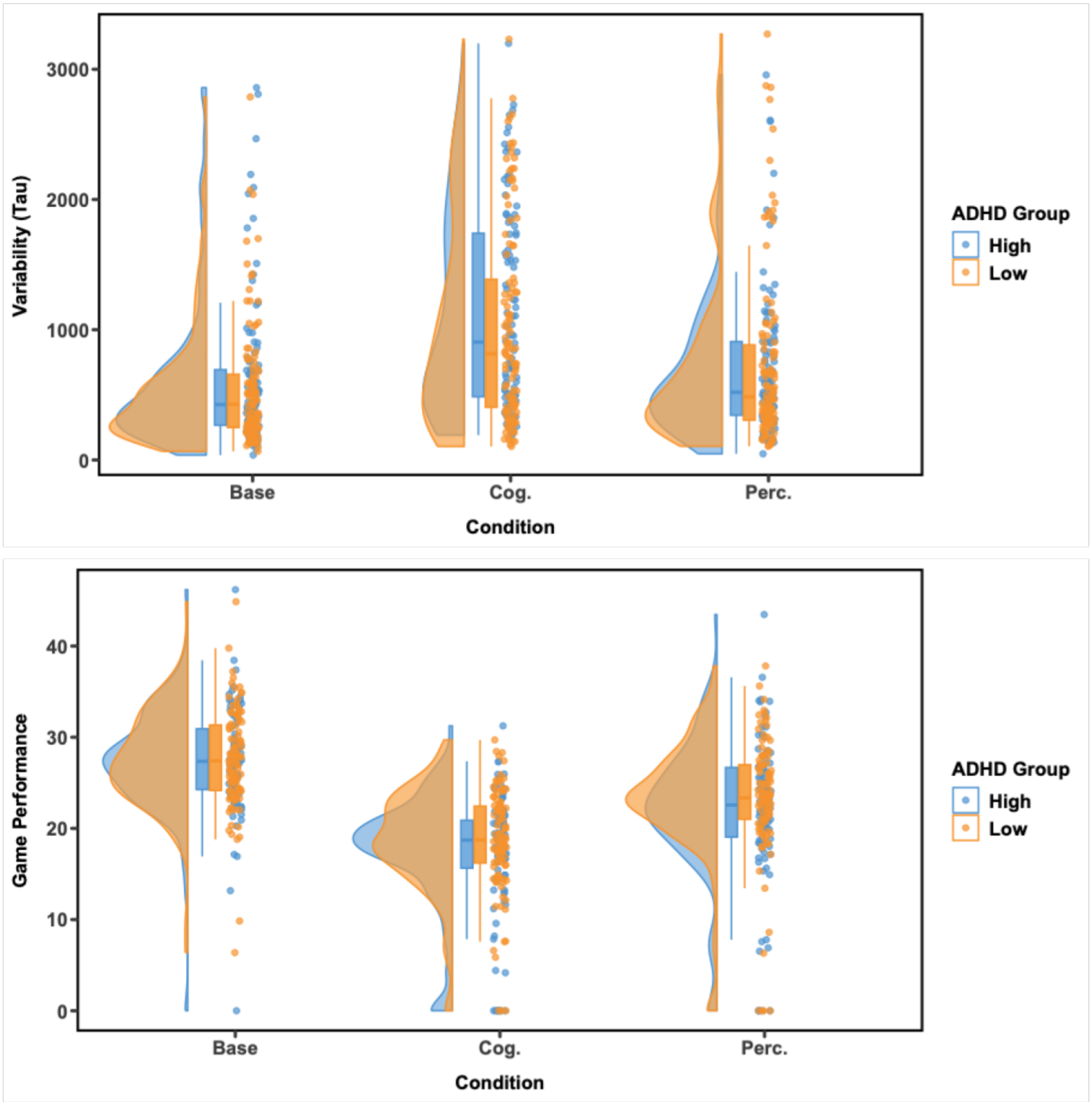

Fig. S3. Raincloud plots of reaction time variability (RTV) and game performance by condition and ADHD status in Experiment 1.

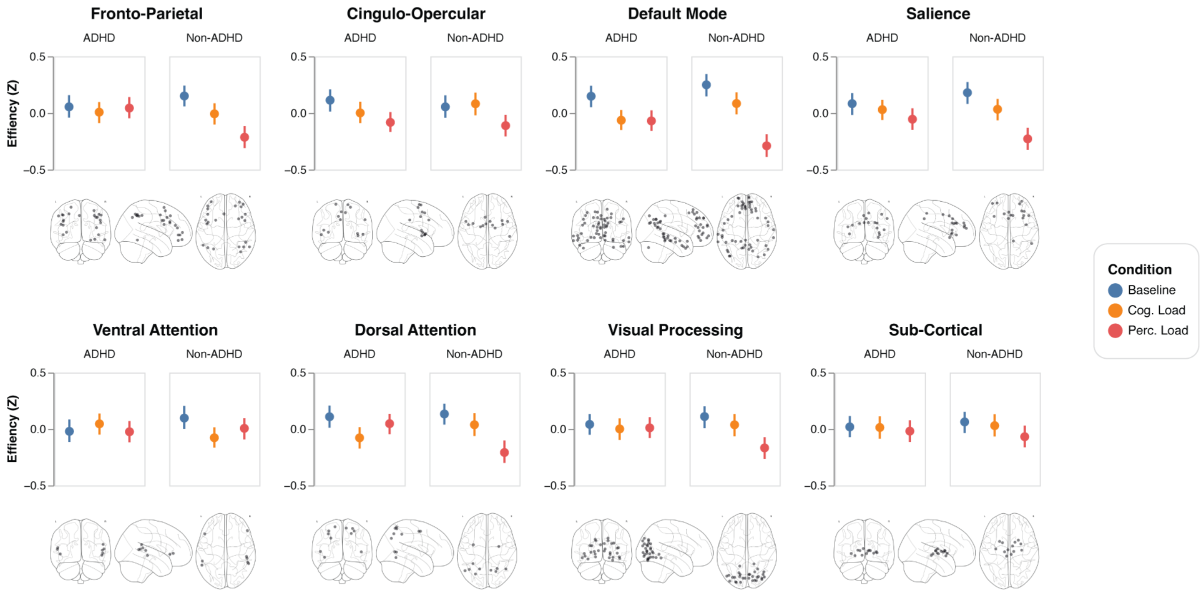

Fig. S4. Brain network efficiency within attention-related subnetworks during the baseline, cognitive load, and perceptual load conditions. Locations of nodes included in each network are plotted below each chart. Error bars represent 95% confidence intervals.

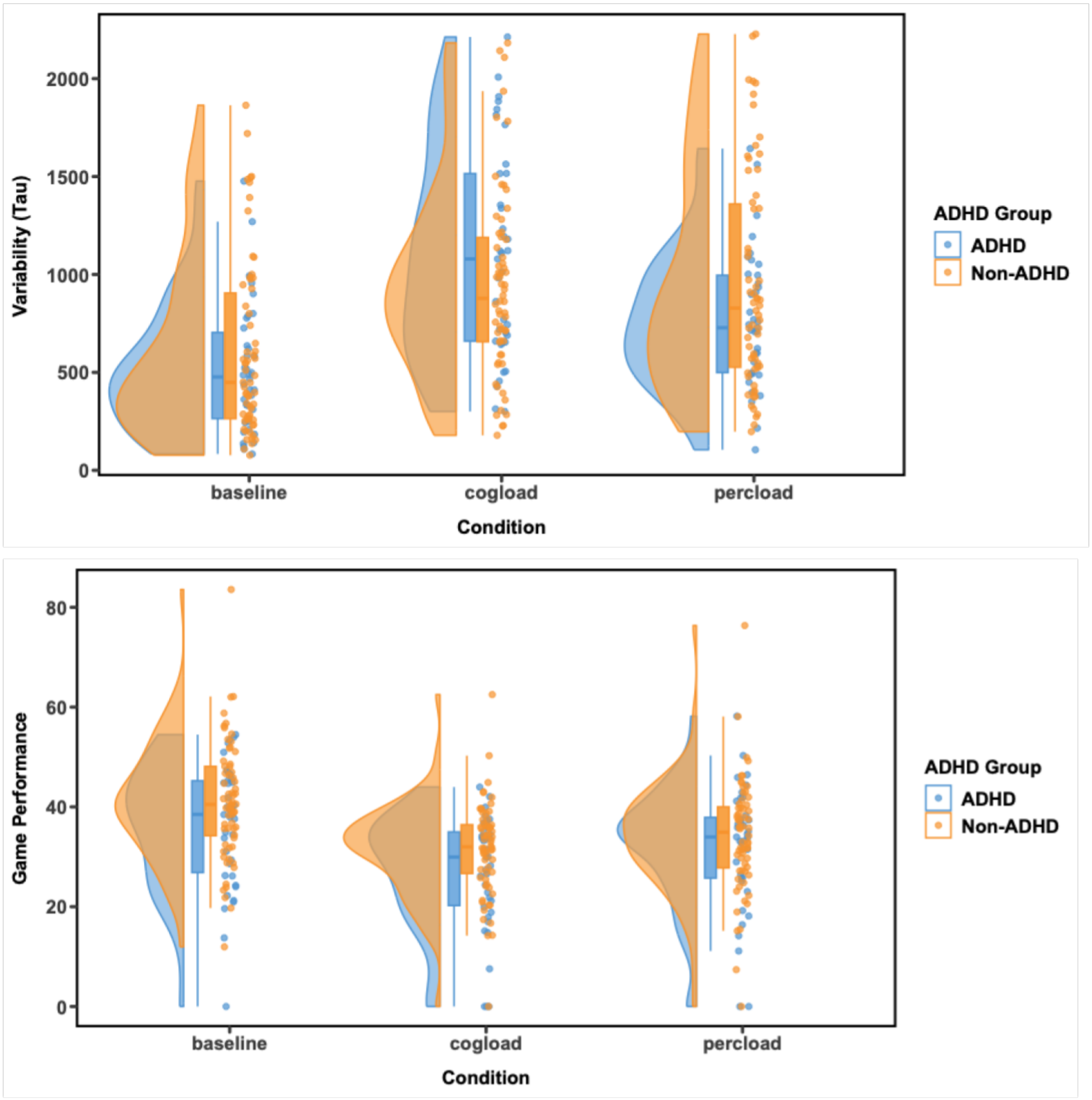

Fig. S5. Raincloud plots of reaction time variability (RTV) and game performance by condition and ADHD status in Experiment 3.

Stimulus

The stimulus used in each of these experiments was *Asteroid Impact*. Asteroid Impact is a point-and-click style video game developed in Python and Unity where subjects use a cursor to collect crystal-shaped targets that are displayed at different locations on the screen while avoiding asteroids that bounce around the screen. Game difficulty is manipulated by altering the number of targets a subject needs to collect, the number of objects to be avoided, and the rate at which these objects move.

The stimulus provides experimental control in that all random aspects of the game are removed; any differences in game experience are the result of player intervention. To help resolve this potential confound, the stimulus provides a high resolution content analysis of all events in the game (e.g., when a crystal is collected, x/y position of the player's cursor, time when player "dies") with 16ms temporal resolution. This content analysis is exported to a .csv file that allows for subsequent computation using a wide variety of analysis packages.

Asteroid Impact Gameplay

In each experiment, some rounds of gameplay contained an additional manipulation designed to induce either cognitive load or perceptual load.

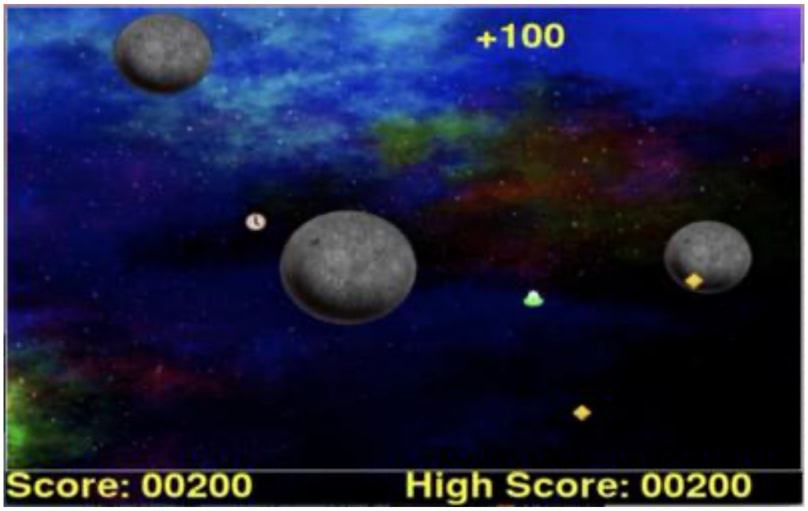

Fig. S6. Depiction of *Asteroid Impact* gameplay. Participants move their cursor (marked by a spaceship icon) around the screen to collect crystals and avoid asteroids.

Cognitive Load

In the Cognitive Load manipulation, participants were told that "some of the crystals are dangerous" and that if they collected two crystals in a row of the same color, they would lose 1000 points (the equivalent of ten crystals). This manipulation was based on a 1-back memory maintenance task (see e.g., Eriksson, Vogel, Lansner, Bergström, & Nyberg, 2015; Veltman, Rombouts, & Dolan, 2003), which has been shown to elicit broad activation in cognitive control-related brain regions and to be perceived cognitively difficult.

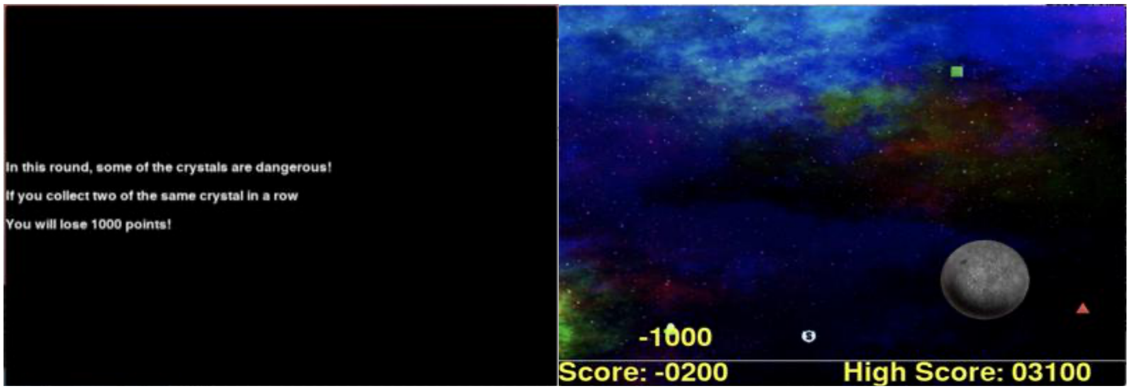

Fig. S7. Depiction of instructions and gameplay for the cognitive load condition of *Asteroid Impact* used in the experiments reported herein.

Perceptual Load
In the Perceptual Load manipulation, in-game objects were obscured through the use of a semi-transparent overlay. Perceptual load was not a topic of interest in this manuscript.

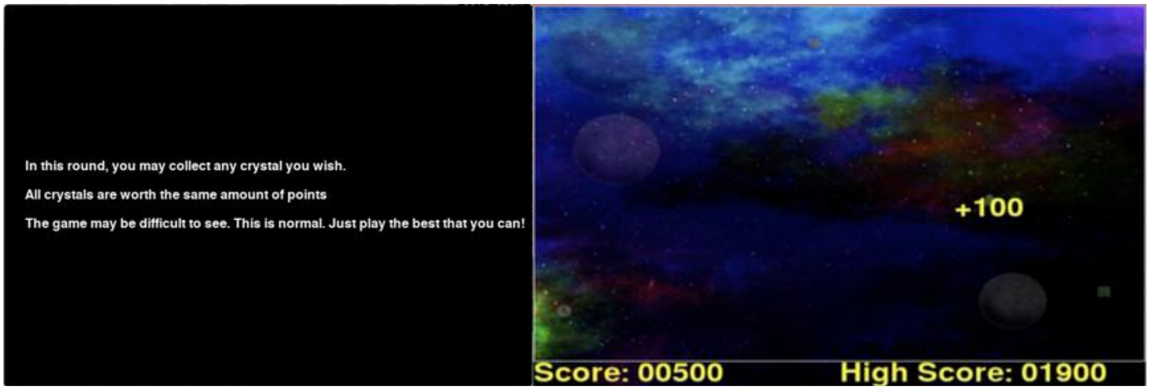

Fig. S8. Depiction of instructions and gameplay for the perceptual load condition of *Asteroid Impact* used in the experiments reported herein.

Reaction Time Task
While completing the *Asteroid Impact* task, participants also completed a reaction time task. In this task, participants were asked to press a key upon seeing a shape appear on screen. Participants were told that they should focus their attention on collecting crystals, but that they should respond as quickly as they can when they see the shape.

Data and Analysis Code
Data and analysis code for the experiments reported herein can be found on the Open Science Framework (https://osf.io/9byvq/).
